# Depression of lncRNA NEAT1 Antagonizes LPS-Evoked Acute Injury and Inflammatory Response in Alveolar Epithelial Cells via HMGB1-RAGE Signaling

**DOI:** 10.1155/2020/8019467

**Published:** 2020-02-05

**Authors:** Hongchao Zhou, Xinhui Wang, Bin Zhang

**Affiliations:** ^1^Department of Intensive Care Units, Zhoukou Central Hospital, Zhoukou, Henan 466000, China; ^2^Department of General Surgery, The First Affiliated Hospital of Xinxiang Medical University, Xinxiang, Henan 453100, China

## Abstract

Sepsis-evoked acute lung injury (ALI) and its extreme manifestation, acute respiratory distress syndrome (ARDS), constitute a major cause of mortality in intensive care units. High levels of the long noncoding RNA nuclear paraspeckle assembly transcript 1 (NEAT1) have been positively correlated with increased severity and unfavorable prognoses in patients with sepsis. Nevertheless, the function and molecular mechanism of NEAT1 in ALI remain elusive. In the current study, high levels of NEAT1 were confirmed in lipopolysaccharide- (LPS-) induced ALI mice models and in LPS-stimulated cells from the alveolar epithelial A549 cell line. Intriguingly, cessation of NEAT1 led to increased cell viability and decreased lactate dehydrogenase release, apoptosis, and caspase-3/9 activity, which conferred protection against LPS-induced injury in these cells. NEAT1 inhibition also restrained LPS-evoked transcripts and production of inflammatory cytokines IL-6, IL-1*β*, and TNF-*α*. A mechanism analysis corroborated the activation of high-mobility group box1 (HMGB1)/receptors for advanced glycation end products (RAGE) and NF-*κ*B signaling in LPS-treated A549 cells. NEAT1 suppression reversed the activation of this pathway. Notably, reactivating HMGB1/RAGE signaling via HMGB1 overexpression blunted the anti-injury and anti-inflammation effects of NEAT1 knockdown. These findings suggest that NEAT1 may aggravate the progression of ALI and ARDS by inducing alveolar epithelial cell injury and inflammation via HMGB1/RAGE signaling, implying a promising treatment target for these conditions.

## 1. Introduction

Infection-evoked sepsis is a life-threatening dysfunctional disease that affects the organs, and the lungs are highly susceptible. Acute lung injury (ALI) is a common clinical symptom in patients with sepsis. Approximately 50% to 55% of patients with ALI will develop severe acute respiratory distress syndrome (ARDS) [1]. ALI/ARDS often occur during clinical complications in intensive care units. The accompanying high-protein pulmonary edema and severe hypoxemic respiratory failure are leading causes of high morbidity and mortality in critically ill patients. Despite progress in understanding the pathogenesis of ALI/ARDS, therapeutic strategies remain unsatisfactory, and mortality remains significant at 35% to 40% [2].

Pulmonary alveolar epithelial cells (AECs) facilitate normal breathing by synthesizing and secreting surfactants. Accumulating evidence has corroborated that the pathophysiological process of ALI is characterized by AEC insult [3, 4]. For example, AEC lesions disrupt cellular integrity and the ability to clear fluid from the alveolar space, clinically translating into lung edema. Intriguingly, recent studies also have defined ALI/ARDS as a syndrome of diffuse inflammatory lung injury [5, 6]. During ALI/ARDS, AECs participate in the inflammatory response by acting as major sources of inflammatory cytokines (e.g., IL-1*β*, IL-6) and chemokines (e.g., MCP-1, IL-8). Recently, considerable interest has focused on the prospective therapeutic value of targeting inflammation and AEC injury in ALI/ARDS [7, 8].

Long noncoding RNAs (lncRNAs) are a class of non-protein-coding RNAs with lengths exceeding 200 nucleotides and limited protein-coding capability. Recently, lncRNA nuclear paraspeckle assembly transcript 1 (NEAT1) has become a subject of interest due to its pleiotropic function in diseases. NEAT1's role in cancer progression has been studied [9, 10]. Increasing research also has implicated NEAT1 in inflammation-related diseases. For instance, upregulation of NEAT1 has been observed in rats with chronic constriction injury, and its knockdown suppresses neuroinflammation and neuropathic pain behaviors [11]. In contrast, NEAT1 affords antiapoptotic and anti-inflammatory functions in traumatic brain injury [12]. Recent evidence has confirmed that circulating NEAT1 is positively related to the risk, severity, and unfavorable prognosis in patients with sepsis [13]. Nevertheless, the role of NEAT1 in sepsis-induced ALI remains elusive.

Thus, in the present study, we examined NEAT1 expression in an animal model of lipopolysaccharide- (LPS-) induced ALI. We also examined its effects on injury and inflammation in an *in vitro* AEC model of LPS-induced ALI.

## 2. Materials and Methods

### 2.1. Animal Model of Sepsis-Induced ALI

Male C57BL/6 mice (6-8 weeks old) weighing 20 to 24 g were purchased from the Vital River Laboratory Animal Technology Co., Ltd (Beijing, China). Before the experiments, all animals were acclimatized for 1 week and fed with food and water *ad libitum*. To construct the model of sepsis-evoked ALI, ten mice in each group were anesthetized via an intraperitoneal injection of 1% pentobarbital and then intravenously injected with LPS (5 mg/kg, Sigma-Aldrich, Taufkirchen, Germany) in 100 *μ*l of PBS, as previously described [14, 15]. The mice in the control group received the same treatment but with no LPS instillation. All surgical and care procedures were conducted according to the National Institutes of Health *Guide for the Care and Use of Laboratory Animals* and approved by the Institutional Animal Care and Use Committee of the First Affiliated Hospital of Xinxiang Medical University. Animals were sacrificed by cervical dislocation. At 2 days after LPS inhalation, lung tissues were collected and frozen in liquid nitrogen. All specimens were stored at -80°C for the subsequent experiments.

### 2.2. Histopathological Evaluation

Lung tissues collected from mice were fixed in 4% PFA for 16 h at room temperature. After being dehydrated by grading ethanol and paraffin embedded, tissue sections (5 *μ*m) were cut and stained with Hematoxylin-Eosin (H&E). The pathological changes in the lung tissues were examined under a light microscope.

### 2.3. Cell Culture and LPS Exposure

Human pulmonary epithelial cell line A549 was acquired from American Type Culture Collection (ATCC; Manassas, VA, USA). Cells were maintained in DMEM supplemented with penicillin (10 U/ml), streptomycin (10 *μ*g/ml), and 10% fetal bovine serum. For LPS exposure, cells were cultured in medium with various doses of LPS (0.1 *μ*g, 1 *μ*g, 10 *μ*g, or 100 *μ*g) for 4 h, 8 h, 12 h, and 24 h. All cells were housed in a sterile incubator containing 5% CO_2_, 95% air at 37°C.

### 2.4. Knockdown of NEAT1 by Lentiviral Vector Infection

The shRNA sequences of NEAT1 and the scramble control (NC) were synthesized by GenePharma (Shanghai, China) and ligated into pGLV3-GFP lentiviral (LV) plasmids. Then, the pGLV3-sh-NEAT1 was transfected into 293T cells with pCMV-VSV-G plasmids to amplify the lentiviruses. Virus purification was conducted using a 0.45 *μ*m filter. The viral titer was assessed using a p24 ELISA kit (Cell Biolabs, Inc., San Diego, CA, USA). For cell infection, A549 cells were infected for 48 h with the LV-sh-NEAT1 or LV-sh-NC. The effects on NEAT1 expression were subsequently assessed by qRT-PCR. For the NEAT1 knockdown in vivo, mice were infected with 1 × 10^8^ PFUs LV-sh-NEAT1 (in 50 *μ*l PBS) through intratracheal injection 7 days prior to LPS treatment.

### 2.5. HMGB1 Overexpression and Knockdown

For the construction of HMGB1 recombinant vector, TRIzol Reagent (Invitrogen, Carlsbad, CA, USA) was applied to extract total RNA from A549 cells. Then, the obtained RNA was converted into the first-strand cDNA using the SuperScript II First-Strand Synthesis System (Invitrogen). Full-length human HMGB1 cDNA was amplified via PCR. After digestion with restriction enzyme, HMGB1 cDNA was inserted into pcDNA3.1(+) plasmids (Invitrogen) to prepare the recombinant pcDNA-HMGB1 vectors. A549 cells with 70% to 80% confluence were then transfected with pCDNA-HMGB1 to overexpress HMGB1 using the Lipofectamine 2000 (Invitrogen). The efficacy of HMGB1 expression was analyzed by western blotting.

The HMGB1-siRNA and the scramble control were synthesized by GenePharma (Shanghai, China). For HMGB1 knockdown, A549 cells with 70% to 80% confluence were then transfected with HMGB1-siRNA using the Lipofectamine 2000 (Invitrogen). The efficacy of HMGB1 expression was analyzed by western blotting.

### 2.6. Quantitative Reverse Transcription Polymerase Chain Reaction (qRT-PCR) Analysis

Total RNA from cells and tissues was prepared and synthesized into cDNA. Then, qRT-PCR was performed to detect expression of NEAT1 using a SYBR® Premix Ex Taq™ II Kit (Takara, Otsu, Japan). The NEAT1-specific primers were designed as previously described [16] and obtained by Shanghai ShengGong Biological Company (Shanghai, China). Primers for the other genes were as follows: IL-6, forward 5′-CCAGGAGAAGATTCCAAAGATGTA-3′, reverse 5′-CGTCGAGGATGTACCGAATTT-3′; IL-1*β*, forward 5′-CTCTCACCTCTCCTACTCACTT-3′, reverse 5′-TCAGAATGTGGGAGCGAATG-3′; and TNF-*α*, forward 5′-GGATGGATGGAGGTGAAAGTAG-3′, reverse 5′-TGATCCTGAAGAGGAGAGAGAA-3′. To quantify the expression of target genes, *β*-actin was used as an internal reference. All specimens were analyzed using an Applied Biosystems 7300 Real-Time PCR System (Applied Biosystems, Foster City, CA, USA). The results were calculated using the 2^-*ΔΔ*Ct^ equation.

### 2.7. Evaluation of Cell Viability by MTT

Cell viability was assessed according to previous methods [17]. Briefly, after washing with PBS, cells seeded in 96-well plates were incubated with DMEM supplemented with MTT (3-(4,5-dimethylthiazol-2-yl)-2,5-diphenyltetrazolium bromide) solution (5 mg/ml, Sigma) for 4 h at 37°C. Subsequently, 200 *μ*l of dimethyl sulfoxide solution was pipetted into each well to dissolve the crystal formazan. Absorbance was captured at 550 nm using a microplate reader (Bio-Rad, Hercules, CA, USA) to evaluate cell viability.

### 2.8. Detection of Cell Apoptosis by Flow Cytometry

After transfection with LV-sh-NEAT1, recombinant HMGB1 vectors, or HMGB1-siRNA, cells were exposed to LPS for 12 h and then collected and fixed with 70% ethanol. Apoptosis was subsequently evaluated by double staining with 10 *μ*l of Annexin V-FITC and 5 *μ*l of PI in the dark for 15 min. All protocols were conducted in accordance with an Annexin-V-FITC Apoptosis Detection Kit (Beyotime, Shanghai, China). To quantify apoptosis, cells were analyzed via flow cytometry (BD Biosciences, San Jose, CA, USA). Both early apoptotic (Annexin V^+^/PI^−^) and late apoptotic (Annexin V^+^/PI^+^) cells were included in cell death determinations.

### 2.9. Analysis of Lactate Dehydrogenase Release

Cells with different treatments were collected and incubated for 2 h in medium supplemented with 150 *μ*l of lactate dehydrogenase (LDH) release reagent. After 5 min of centrifugation, concentrations of LDH in the supernatants were measured according to the standard protocols of a commercial LDH Detection Kit (Beyotime).

### 2.10. Measurement of Caspase-3 and Caspase-9 Activity

The activities of caspase-3 and caspase-9 were evaluated using Caspase-3 Fluorescent Assay Kits and Caspase-9 Fluorescence Assay Kits (Clontech, Palo Alto, CA), respectively, according to the company's specifications. Briefly, cells under various conditions were lysed with lysis buffer and then centrifuged at 4°C to prepare the supernatants. Then, the reaction buffer containing 5 *μ*l of the specific substrate of DEVD-AFC for caspase-3 or LEHD-AMC for caspase-9 was supplemented into supernatants and incubated for 1 h at 37°C. All specimens were subsequently analyzed using an FL600 fluorescent plate reader (BioTek Instruments, VT, USA) to determine the fluorescent intensity.

### 2.11. ELISA for Inflammatory Cytokines

Following treatment with the indicated conditions, cells were collected, and the concentrations of IL-6, IL-1*β*, and TNF-*α* in the culture medium were measured using commercial ELISA Kits (Invitrogen). All protocols were conducted according to manufacturer instructions.

### 2.12. Western Blotting

An ice-cold RIPA buffer was used to extract total protein that was quantified using a BCA Protein Detection Kit (Pierce, Rockford, IL, USA). Then, equal amounts of protein were subjected to 12% SDS-PAGE, following transfer to nitrocellulose membranes. Nonspecific binding was interdicted using 5% nonfat milk. The membranes were incubated at 4°C overnight with primary antibodies against HMGB-1 (1 : 30000), receptors for advanced glycation end products (RAGE, 1 : 4000), and p-p65NF-*κ*B (1 : 3000), all from Abcam (Cambridge, UK, USA). Following incubation with horseradish peroxidase-conjugated secondary antibodies, membranes were treated with ECL Reagent (Beyotime) to visualize the immunoreactive bands. The bands were quantified using a Gel Doc™ XR imaging system (Bio-Rad Laboratories, Hercules, CA, USA) and ImageJ software. For normalization, *β*-actin was enrolled as an internal standard.

### 2.13. Statistical Analysis

All experiments were performed at least three times. Statistical calculations were analyzed using SPSS 19.0 software (SPSS Inc., Chicago, IL, USA). Data are shown as the mean ± standard deviation (SD). Differences between groups were tested using Student's *t*-test for two groups and one-way ANOVA or two-way ANOVA for three or more groups, concomitant with subsequent analysis via post hoc Student-Newman-Keuls test. *P* < 0.05 was considered statistically significant.

## 3. Results

### 3.1. High Expression of lncRNA NEAT1 Was Observed in a Mouse Model of Acute Lung Injury and in LPS-Injured Alveolar Epithelial Cells

**Figure 1:**
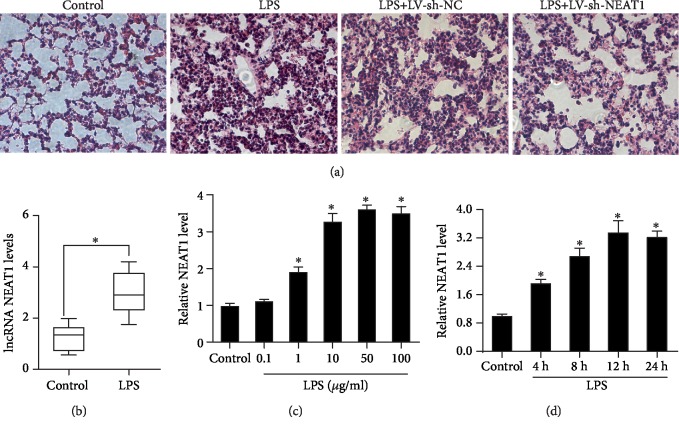
High levels of NEAT1 were observed in AEC and mouse models of LPS-induced acute lung injury (ALI). (a) Mice were injected with LPS to construct a sepsis-induced ALI model. To downregulate the expression of NEAT1, mice were infected by LV-sh-NEAT1 or LV-sh-NC prior to LPS treatment. Histological changes in the lung tissues were observed by H&E staining (×200). (b) The levels of NEAT1 then were detected using qRT-PCR. Six mice were used in each group. (c) A549 cells were treated with various doses of LPS for 12 h, and the levels of NEAT1 were then analyzed (*n* = 3). (d) Following exposure to LPS (10 *μ*g/ml) for the indicated times, the levels of NEAT1 in A549 cells were determined (*n* = 3); ^∗^*P* < 0.05.

To explore how NEAT1 affects sepsis-evoked ALI, we constructed a mouse model of ALI via LPS injection *in vivo*. Lung tissues were collected after LPS treatment for H&E staining. The lung tissues from LPS-injured mice showed obvious pathological changes, such as thickening of the alveolar wall, lung edema, pulmonary congestion, and alveolar hemorrhage (Figure 1(a)). NEAT1 knockdown by LV-sh-NEAT1 infection significantly alleviated the lung tissue injury induced by LPS (Figure 1(a)). Higher levels of NEAT1 were observed in the lung tissues from LPS-injured mice relative to control groups (Figure 1(b)). We further simulated ALI *in vitro* by exposing A549 pulmonary epithelial cells to LPS, and the results corroborated that treatment with 1 *μ*g of LPS dramatically increased NEAT1 expression (Figure 1(c)). Moreover, we observed dose-dependent elevation in NEAT1 expression following LPS exposure and no observable difference in the groups that were treated with 10 *μ*g or 50 *μ*g of LPS (Figure 1(c)). Additionally, treatment with 10 *μ*g of LPS enhanced the expression of NEAT1 in a time-dependent manner. The peak value was observed at 12 h after challenge (Figure 1(d)).

### 3.2. NEAT1 Knockdown Ameliorated LPS-Induced Alveolar Epithelial Cell Injury

**Figure 2:**
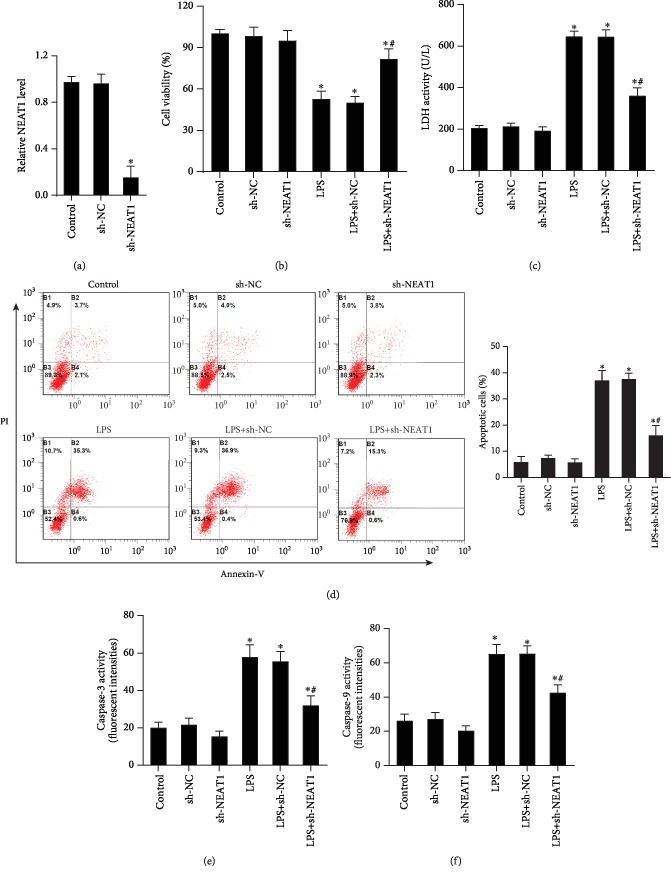
Cessation of NEAT1 antagonized cell injury in LPS-exposed A549 cells. (a) A549 cells were infected with LV-sh-NEAT1, and the levels of NEAT1 were evaluated using a qRT-PCR assay (*n* = 3). (b) Cell viability was measured via MTT analysis (*n* = 3). (c) Lactate dehydrogenase (LDH) release, (d) cell apoptosis, and (e) caspase-3 and (f) caspase-9 levels were detected in A549 cells (*n* = 3). ^∗^*P* < 0.05 vs. the control group. ^#^*P* < 0.05 vs. the LPS-exposed group.

As shown in Figure 2(a), infection with LV-NEAT1 plasmids notably suppressed the expression of NEAT1 in A549 cells as compared to the control group (*P* < 0.05). A two-way ANOVA was performed to detect the effect of LPS stimulation and NEAT1 knockdown in Figure 2. The result showed that LPS stimulation and NEAT1 knockdown had a significant effect on cell viability ((*F*(1, 12) = 155, *P* < 0.05), (*F*(2, 12) = 9.148, *P* < 0.05), respectively), LDH production ((*F*(1, 12) = 677.5, *P* < 0.05), (*F*(2, 12) = 56.52, *P* < 0.05), respectively), apoptosis ((*F*(1, 12) = 328.8, *P* < 0.05), (*F*(2, 12) = 31.49, *P* < 0.05), respectively), and activity of caspase-3 ((*F*(1, 12) = 165.6, *P* < 0.05), (*F*(2, 12) = 19.31, *P* < 0.05), respectively) and caspase-9 ((*F*(1, 12) = 250.2, *P* < 0.05), (*F*(2, 12) = 21.29, *P* < 0.05), respectively). The interaction of LPS and NEAT1 knockdown also had a significant effect on cell viability (*F*(2, 12) = 15.6, *P* < 0.05), LDH production (*F*(2, 12) = 45.36, *P* < 0.05), apoptosis (*F*(2, 12) = 26.78, *P* < 0.05), and activity of caspase-3 (*F*(2, 12) = 8.11, *P* < 0.05) and caspase-9 (*F*(2, 12) = 6.835, *P* < 0.05).

NEAT1 knockdown without LPS stimulation did not alter cell viability obviously, LDH production, apoptosis, and activity of caspase-3 and caspase-9, as compared to the control group (Figures 2(b)–2(f)). This might be due to the basic low levels of NEAT1 in the A549 cells. LPS-exposed cells exhibited low viability, which was revered by NEAT1 knockdown (Figure 2(b)). Simultaneously, LPS treatment led to increased LDH release, which is a marker for cell injury. NEAT1 knockdown antagonized this increase in LPS-simulated A549 cells (Figure 2(c)). Furthermore, NEAT1 silencing attenuated LPS-induced apoptosis (Figure 2(d)). Meanwhile, LPS exposure caused a notable elevation in caspase-3 (Figure 2(e)) and caspase-9 (Figure 2(f)) activity in A549 cells. Intriguingly, these increases were abrogated when cells were pretreated with the LV-sh-NEAT1.

### 3.3. NEAT1 Knockdown Suppressed Inflammation in LPS-Stimulated Alveolar Epithelial Cells

**Figure 3:**
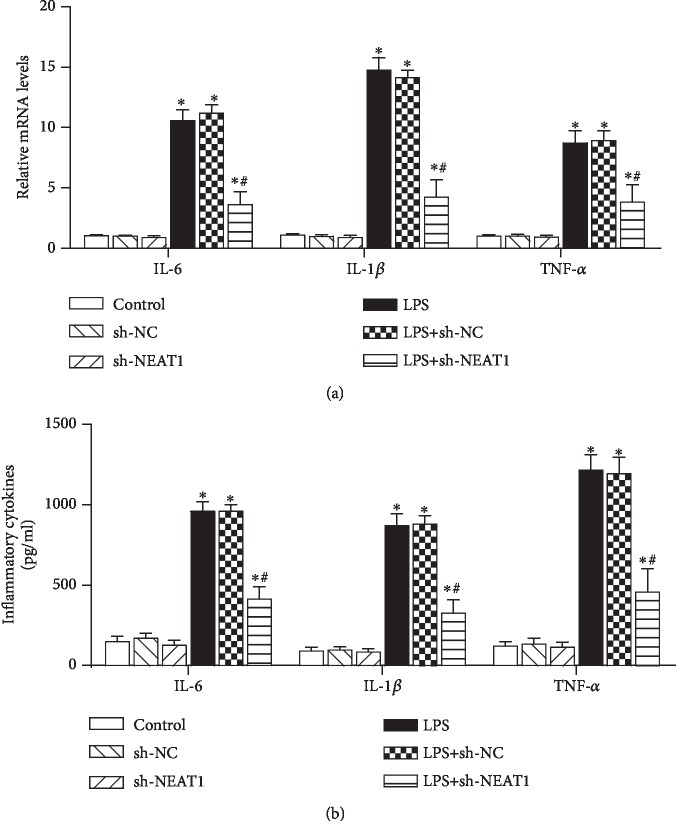
NEAT1 suppression restrained LPS-evoked inflammation in A549 cells. After infection with LV-sh-NEAT1 or LV-sh-NC, A549 cells were exposed to LPS. (a) The mRNA levels of IL-6, IL-1*β*, and TNF-*α* were analyzed using a qRT-PCR assay (*n* = 3). (b) An ELISA was conducted to measure the production of IL-6, IL-1*β*, and TNF-*α* in supernatants of A549 cells (*n* = 3). ^∗^*P* < 0.05 vs. the control group. ^#^*P* < 0.05 vs. the LPS-treated group.

Excessive lung inflammation is a proverbial feature of sepsis-related ALI/ARDS [6, 18]. Therefore, we elucidated the roles of NEAT1 in LPS-induced inflammation in AECs. A two-way ANOVA was performed to detect the effect of LPS stimulation and NEAT1 knockdown on inflammation. The result showed that LPS stimulation, NEAT1 knockdown, and the interaction of LPS and NEAT1 knockdown had a significant effect on transcription of IL-6 ((*F*(1, 12) = 602.5, *P* < 0.05), (*F*(2, 12) = 65.2, *P* < 0.05), (*F*(2, 12) = 61.64, *P* < 0.05), respectively), IL-1*β* ((*F*(1, 12) = 728.4, *P* < 0.05), (*F*(2, 12) = 86.29, *P* < 0.05), (*F*(2, 12) = 81.74, *P* < 0.05), respectively), and TNF-*α* ((*F*(1, 12) = 272.9, *P* < 0.05), (*F*(2, 12) = 20.64, *P* < 0.05), (*F*(2, 12) = 19.2, *P* < 0.05), respectively) in A549 cells (Figure 3(a)). LPS stimulation, NEAT1 knockdown, and the interaction of LPS and NEAT1 knockdown had a significant effect on the production of IL-6 ((*F*(1, 12) = 752.4, *P* < 0.05), (*F*(2, 12) = 70.84, *P* < 0.05), (*F*(2, 12) = 55.31, *P* < 0.05), respectively), IL-1*β* ((*F*(1, 12) = 581.1, *P* < 0.05), (*F*(2, 12) = 55.77, *P* < 0.05), (*F*(2, 12) = 52.21, *P* < 0.05), respectively), and TNF-*α* ((*F*(1, 12) = 413.9, *P* < 0.05), (*F*(2, 12) = 38.47, *P* < 0.05), (*F*(2, 12) = 35.73, *P* < 0.05), respectively) in A549 cells (Figure 3(b)).

As presented in Figure 3, LPS treatment enhanced the transcription and production of IL-6, IL-1*β*, and TNF-*α* in A549 cells, which was reversed by NEAT1 cessation. However, NEAT1 knockdown without LPS stimulation did not alter the transcription and production of IL-6, IL-1*β*, and TNF-*α* obviously, as compared to the control group (Figures 3(a) and 3(b)).

### 3.4. Cessation of NEAT1 Restrained LPS-Evoked Activation of HMGB1-RAGE Signaling

**Figure 4:**
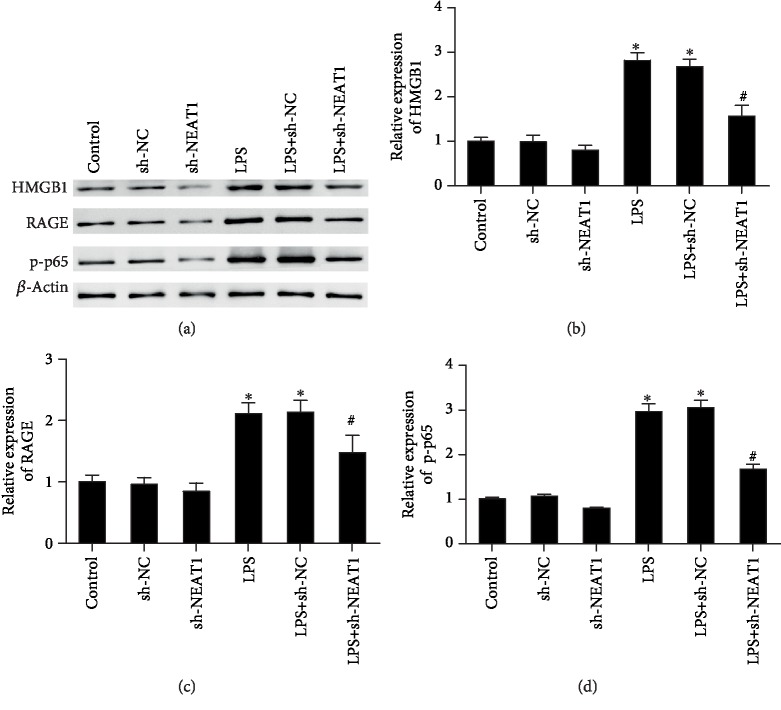
Cessation of NEAT1 inhibited activation of the HMGB1-RAGE signaling in LPS-exposed A549 cells. (a) Following NEAT1 knockdown in A549 cells, cells were exposed to LPS for 12 h. The protein levels of HMGB1 and downstream RAGE and p-p65 NF-*κ*B were analyzed by western blotting (*n* = 3). The corresponding quantified analysis of (b) HMGB1, (c) RAGE, and (d) p-p65 NF-*κ*B was performed using ImageJ software (*n* = 3); ^∗^*P* < 0.05, ^#^*P* < 0.05.

Increasing evidence has confirmed that aberrant activation of the HMGB1/RAGE pathway has a critical role in ALI [19]. We next determined the effect of LPS stimulation and NEAT1 knockdown on the activation of this signaling. A two-way ANOVA showed that LPS stimulation and NEAT1 knockdown had a significant effect on the expression of HMGB1 ((*F*(1, 12) = 336.7, *P* < 0.05), (*F* (2, 12) = 35.29, *P* < 0.05), respectively), RAGE ((*F*(1, 12) = 132.9, *P* < 0.05), (*F*(2, 12) = 9.769, *P* < 0.05), respectively), and p-p65 ((*F*(1, 12) = 259.6, *P* < 0.05), (*F*(2, 12) = 20.4, *P* < 0.05), respectively) in A549 cells (Figure 4). The interaction of LPS and NEAT1 knockdown also had a significant effect on the expression of HMGB1 (*F*(2, 12) = 18.03, *P* < 0.05), RAGE (*F* (2, 12) = 4.2, *P* < 0.05), and p-p65 (*F*(2, 12) = 9.02, *P* < 0.05) in A549 cells (Figure 4).

As exhibited in Figure 4(a), LPS exposure increased expression of HMGB1 and resulted in a 3.12-fold elevation in its protein expression (Figure 4(b)). However, NEAT1 ablation restrained this high expression of HMGB1 that was triggered by LPS (Figures 4(a) and 4(b)). Concomitantly, the expression of downstream RAGE (Figures 4(a) and 4(c)) and p-p65/NF-*κ*B (Figures 4(a) and 4(d)) increased in LPS-exposed cells and decreased in NEAT1 downregulated cells. NEAT1 knockdown without LPS stimulation did not affect the transcription and production of HMGB1, RAGE, and p-p65 obviously, as compared to the control group (Figures 4(a)–4(d)).

### 3.5. Restoring the HMGB1-RAGE Pathway Abrogated the Adverse Effects of NEAT1 Inhibition on Alveolar Epithelial Cell Injury and Inflammation

**Figure 5:**
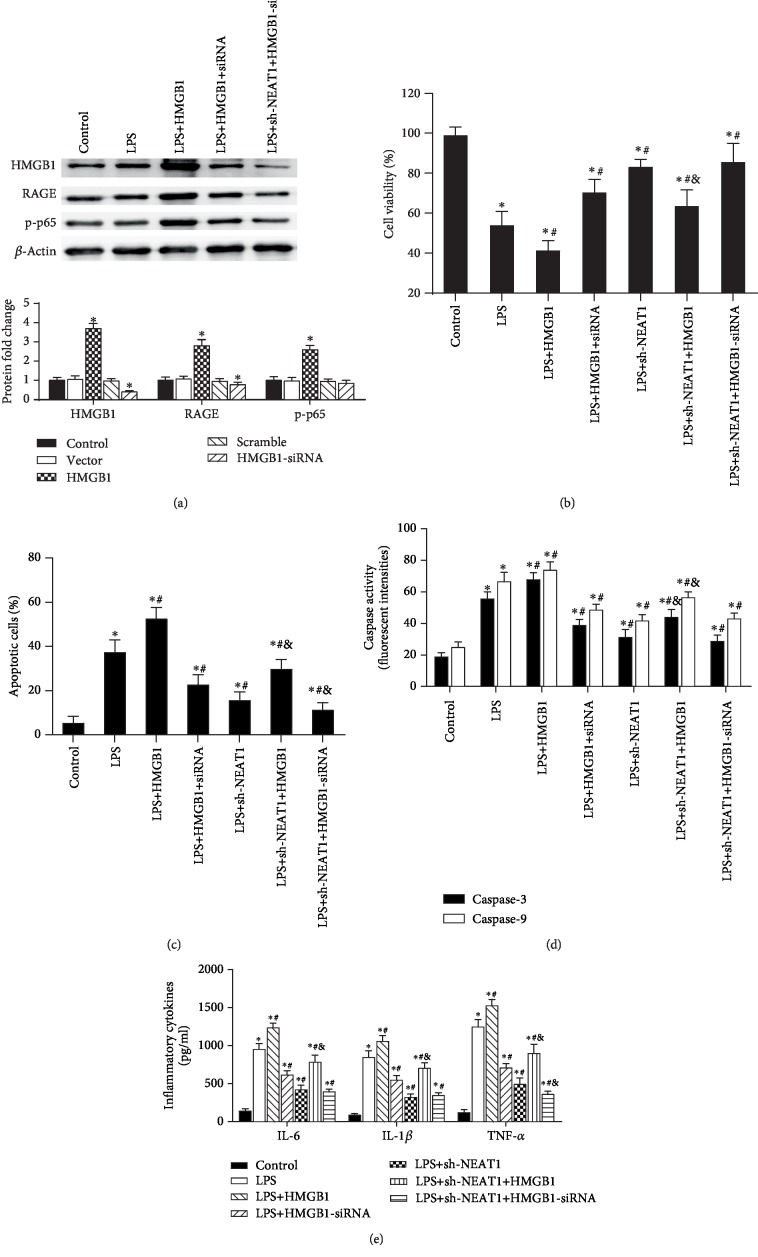
Reactivating HMGB1 signaling overturned the inhibitory functions of NEAT1 cessation on the injury and inflammation of LPS-stimulated A549 cells. (a) Cells were transfected with the recombinant HMGB1 vectors, and the expression of HMGB1, RAGE, and p-p65 NF-*κ*B was assessed using western blot (*n* = 3). After reactivating the HMGB1 signaling by HMGB1 elevation, the effects on (b) cell viability, (c) apoptosis, (d) caspase-3/9 activity, and (e) inflammatory cytokine levels were evaluated in A549 cells (*n* = 3). ^∗^*P* < 0.05 vs. the control group. ^#^*P* < 0.05 vs. the LPS-treated group. ^&^*P* < 0.05 vs. the LPS+sh-NEAT1 groups.

To further clarify the involvement between HMGB1-RAGE signaling and NEAT1-mediated functions in AEC injury and inflammation, we restored or knockdown HMGB1 expression by transfection with recombinant HMGB1 plasmids or HMGB1-siRNA, respectively (Figure 5(a)). Levels of NEAT1 were not noticeably altered by HMGB1 overexpression or knockdown (Supplementary [Supplementary-material supplementary-material-1]). Simultaneously, replenishing HMGB1 increased the expression of RAGE and p-p65NF-*κ*B, and HMGB1 knockdown decreased the expression of RAGE (Figure 5(a)). NEAT1 cessation ameliorated LPS-inhibited cell viability, which was partly reversed by restoring HMGB1/RAGE signaling and little affected by further HMGB1 knockdown (Figure 5(b)). Additionally, HMGB1 overexpression partly abrogated the suppressive effects of NEAT1 knockdown on LPS-evoked apoptosis (Figure 5(c)) and activities of caspase-3 and caspase-9 (Figure 5(d)). Concentrations of proinflammatory cytokines were decreased by NEAT1 knockdown in LPS-exposed A549 cells and were elevated by HMGB1 overexpression (Figure 5(e)). HMGB1 knockdown further decreased the apoptosis and TNF-*α* production, but affected little on caspase-3/caspase-9 activities and production of IL-6 and IL-1*β*, as compared to the LPS+sh-NEAT1 group (Figures 5(c)–5(e)).

## 4. Discussion

ALI and its severe form, ARDS, are potentially fatal complications of sepsis and important cause of high mortality in intensive care units [2]. Recent research highlights the function of lncRNAs in inflammation and injury progression. In the current study, we constructed a mouse model of LPS-induced ALI and observed high expression of the lncRNA NEAT1 in the lung tissues of injured mice. Recent evidence also has confirmed that patients with sepsis tend to have high levels of NEAT1, increased disease severity, and poor prognosis [13]. LPS-induced endotoxemia is a major inducer of ALI; thus, exposing AECs to LPS has been comprehensively applied as an in vitro model to investigate this condition [3, 20]. We corroborated high expression of NEAT1 in LPS-exposed AECs. Together, these data indicate a potential function of NEAT1 in the development of ALI.

Convincing evidence has confirmed that ALI/ARDS is characterized by injury to AECs [3, 4]. Type II AECs are important components of epithelium. They maintain alveolar capillary barrier integrity and produce pulmonary surfactant to decrease lung surface tension. During ALI, surfactant release from AECs is impaired, which blocks alveolar fluid clearance and lowers lung compliance. Protecting AECs from cell injury thus represents a potential therapeutic strategy against sepsis-induced ALI [3, 8]. Therefore, we investigated the effects of NEAT1 on LPS-exposed A549 cells. Intriguingly, inhibition of NEAT1 antagonized the adverse function of LPS on AEC viability. Moreover, NEAT1 cessation restrained LPS-induced LDH release, apoptosis, and caspase-3/9 activity, indicating the protective roles of NEAT1 knockdown in ameliorating LPS-triggered AEC injury and apoptosis. Notably, suppression of NEAT1 also blunts myocardial ischemia reperfusion injury by abrogating apoptosis in diabetic rats [21]. Inversely, elevation of NEAT1 via bexarotene facilitates functional recovery in mice with traumatic brain injury by inhibiting cell apoptosis and inflammation [12].

Accumulating evidences confirm abundant inflammatory cell infiltration and inflammatory cytokine production in ALI/ARDS as major causes for lung function decline and death [5, 22]. Lowering inflammation thus may be a promising therapeutic approach in sepsis-evoked ALI/ARDS [5, 7]. Analogous with previous study [23], LPS exposure also increased releases of proinflammatory cytokines IL-6, IL-1*β*, and TNF-*α* in A549 cells. We next explored whether NEAT1 played roles in LPS-triggered inflammation in AECs. As expected, abrogation of NEAT1 suppressed LPS-induced transcription and production of IL-6, IL-1*β*, and TNF-*α*, indicating a possible proinflammatory function of NEAT1 in ALI. Intriguingly, NEAT1 has been recently identified as a novel inflammatory regulator in human lupus [24]. Additionally, blocking NEAT1 expression may treat inflammatory bowel disease by suppressing inflammatory progression [25]. The anti-inflammatory efficacy of NEAT1 also has been demonstrated in mice with traumatic brain injury [12].

We next clarified the mechanism underlying NEAT1-mediated functions in LPS-stimulated A549 cells and corroborated that exposure to LPS activated the HMGB1/RAGE-NF-*κ*B pathway. HMGB1 has been involved in various cells and in many physiological processes, such as cell growth, migration, and inflammatory response. Recent studies have documented that HMGB1 fulfills critical roles in injury-related diseases by binding with its specific receptor, RAGE [26, 27]. HMGB1 can act as a bona fide, targetable, damage-associated molecular pattern that selectively triggers a neutrophil-mediated injury amplification loop necrosis [26]. Emerging evidence confirms the excessive activation of HMGB1/RAGE signaling in sepsis models and in patients with sepsis [19, 28]. In this study, restoring the HMGB1-RAGE pathway reversed the anti-injury and anti-inflammation efficacy of NEAT1 knockdown in LPS-exposed AECs. Intriguingly, blocking the HMGB1/RAGE axis may ameliorate sepsis-evoked mortality and ALI in mice by attenuating inflammation [29]. In a translational mouse model of ARDS, targeting RAGE exhibits potential therapeutic effects by decreasing alveolar inflammation and apoptosis [28]. Unfortunately, the target gene of NEAT1 was not verified in the present study. HMGB1 or RAGE was not the predicted target of NEAT1, as analyzed by the Starbase (http://starbase.sysu.edu.cn/index.php). So, NEAT1 might indirectly affect the activity and expression of HMGB1-RAGE through other target genes or miRNA.

## 5. Conclusions

Collectively, this research highlights the high expression of NEAT1 in a mouse model and AEC model of LPS-induced ALI. Importantly, cessation of NEAT1 afforded protective effects against LPS-induced AEC injury and inflammation by suppressing HMGB1/RAGE-NF-*κ*B signaling. These findings indicate that NEAT1 aggravates sepsis-evoked ALI/ARDS by inducing AEC injury and inflammation. NEAT1 thus provides an attractive target for developing therapeutic strategy for ALI/ARDS.

## Data Availability

The data used to support the findings of this study are included within the article.

## Supplementary Materials

Supplementary Materials Supplementary Figure 1: NEAT1 levels were detected using RT-PCR. Cells were transfected with HMGB1 overexpression vector or HMGB1-siRNA. ^∗^*P* < 0.05 vs. the control group.

## References

[1] Audimoolam V. K., McPhail M. J., Wendon J. A. (2014). Lung injury and its prognostic significance in acute liver failure. *Critical Care Medicine*.

[2] Avecillas J. F., Freire A. X., Arroliga A. C. (2006). Clinical epidemiology of acute lung injury and acute respiratory distress syndrome: incidence, diagnosis, and outcomes. *Clinics in Chest Medicine*.

[3] Tojo K., Tamada N., Nagamine Y., Yazawa T., Ota S., Goto T. (2018). Enhancement of glycolysis by inhibition of oxygen-sensing prolyl hydroxylases protects alveolar epithelial cells from acute lung injury. *The FASEB Journal*.

[4] Fanelli V., Ranieri V. M. (2015). Mechanisms and clinical consequences of acute lung injury. *Annals of the American Thoracic Society*.

[5] Zhao D., Ding R., Mao Y., Wang L., Zhang Z., Ma X. (2012). Heparin rescues sepsis-associated acute lung injury and lethality through the suppression of inflammatory responses. *Inflammation*.

[6] Reiss L. K., Schuppert A., Uhlig S. (2018). Inflammatory processes during acute respiratory distress syndrome: a complex system. *Current Opinion in Critical Care*.

[7] Deng G., He H., Chen Z. (2017). Lianqinjiedu decoction attenuates LPS-induced inflammation and acute lung injury in rats via TLR4/NF-*κ*B pathway. *Biomedicine & Pharmacotherapy*.

[8] Li C., Huang Y., Yao X. (2016). Lugrandoside attenuates LPS-induced acute respiratory distress syndrome by anti-inflammation and anti-apoptosis in mice. *American Journal of Translational Research*.

[9] Wang H., Yu Y., Fan S., Luo L. (2017). Knockdown of long non-coding RNA NEAT1 inhibits proliferation and invasion and induces apoptosis of osteosarcoma by inhibiting miR-194 expression. *Yonsei Medical Journal*.

[10] Yu X., Li Z., Zheng H., Chan M. T., Wu W. K. (2017). NEAT1: a novel cancer-related long non-coding RNA. *Cell Proliferation*.

[11] Xia L. X., Ke C., Lu J. M. (2018). NEAT1 contributes to neuropathic pain development through targeting miR-381/HMGB1 axis in CCI rat models. *Journal of Cellular Physiology*.

[12] Zhong J., Jiang L., Huang Z. (2017). The long non-coding RNA Neat1 is an important mediator of the therapeutic effect of bexarotene on traumatic brain injury in mice. *Brain, Behavior, and Immunity*.

[13] Huang Q., Huang C., Luo Y., He F., Zhang R. (2018). Circulating lncRNA NEAT1 correlates with increased risk, elevated severity and unfavorable prognosis in sepsis patients. *The American Journal of Emergency Medicine*.

[14] Tu G. W., Shi Y., Zheng Y. J. (2017). Glucocorticoid attenuates acute lung injury through induction of type 2 macrophage. *Journal of Translational Medicine*.

[15] Stephens R. S., Johnston L., Servinsky L., Kim B. S., Damarla M. (2015). The tyrosine kinase inhibitor imatinib prevents lung injury and death after intravenous LPS in mice. *Physiological Reports*.

[16] Gong W., Zheng J., Liu X., Ma J., Liu Y., Xue Y. (2016). Knockdown of NEAT1 restrained the malignant progression of glioma stem cells by activating microRNA let-7e. *Oncotarget*.

[17] Sun L., Pan S., Yang Y. (2016). Toll-like receptor 9 regulates melanogenesis through NF-*κ*B activation. *Experimental Biology and Medicine*.

[18] Yang R., Zou X., Tenhunen J., Tonnessen T. I. (2017). HMGB1 and extracellular histones significantly contribute to systemic inflammation and multiple organ failure in acute liver failure. *Mediators of Inflammation*.

[19] Li K., Yang J., Han X. (2016). Ketamine attenuates sepsis-induced acute lung injury via regulation of HMGB1-RAGE pathways. *International Immunopharmacology*.

[20] Li S., Guo L., Qian P. (2015). Lipopolysaccharide induces autophagic cell death through the PERK-dependent branch of the unfolded protein response in human alveolar epithelial A549 cells. *Cellular Physiology and Biochemistry*.

[21] Ma M., Hui J., Zhang Q. Y., Zhu Y., He Y., Liu X. J. (2018). Long non-coding RNA nuclear-enriched abundant transcript 1 inhibition blunts myocardial ischemia reperfusion injury via autophagic flux arrest and apoptosis in streptozotocin-induced diabetic rats. *Atherosclerosis*.

[22] Hu H., Shi D., Hu C., Yuan X., Zhang J., Sun H. (2017). Dexmedetomidine mitigates CLP-stimulated acute lung injury via restraining the RAGE pathway. *American Journal of Translational Research*.

[23] Hecker M., Behnk A., Morty R. E. (2015). PPAR-*α* activation reduced LPS-induced inflammation in alveolar epithelial cells. *Experimental Lung Research*.

[24] Zhang F., Wu L., Qian J. (2016). Identification of the long noncoding RNA NEAT1 as a novel inflammatory regulator acting through MAPK pathway in human lupus. *Journal of Autoimmunity*.

[25] Liu R., Tang A., Wang X. (2018). Inhibition of lncRNA NEAT1 suppresses the inflammatory response in IBD by modulating the intestinal epithelial barrier and by exosome-mediated polarization of macrophages. *International Journal of Molecular Medicine*.

[26] Huebener P., Pradere J. P., Hernandez C. (2015). The HMGB1/RAGE axis triggers neutrophil-mediated injury amplification following necrosis. *The Journal of Clinical Investigation*.

[27] Weber D. J., Gracon A. S., Ripsch M. S. (2014). The HMGB1-RAGE axis mediates traumatic brain injury–induced pulmonary dysfunction in lung transplantation. *Science Translational Medicine*.

[28] Blondonnet R., Audard J., Belville C. (2017). RAGE inhibition reduces acute lung injury in mice. *Scientific Reports*.

[29] Wang Q., Wu X., Tong X., Zhang Z., Xu B., Zhou W. (2015). Xuebijing ameliorates sepsis-induced lung injury by downregulating HMGB1 and RAGE expressions in mice. *Evidence-based Complementary and Alternative Medicine*.

