# Supplementary figures and images for: Depression of lncRNA NEAT1 Antagonizes LPS-Evoked Acute Injury and Inflammatory Response in Alveolar Epithelial Cells via HMGB1-RAGE Signaling
Source: Mediators Inflamm. 2020 Feb 5;2020:8019467. doi: 10.1155/2020/8019467 (PMC7025070; doi:10.1155/2020/8019467)

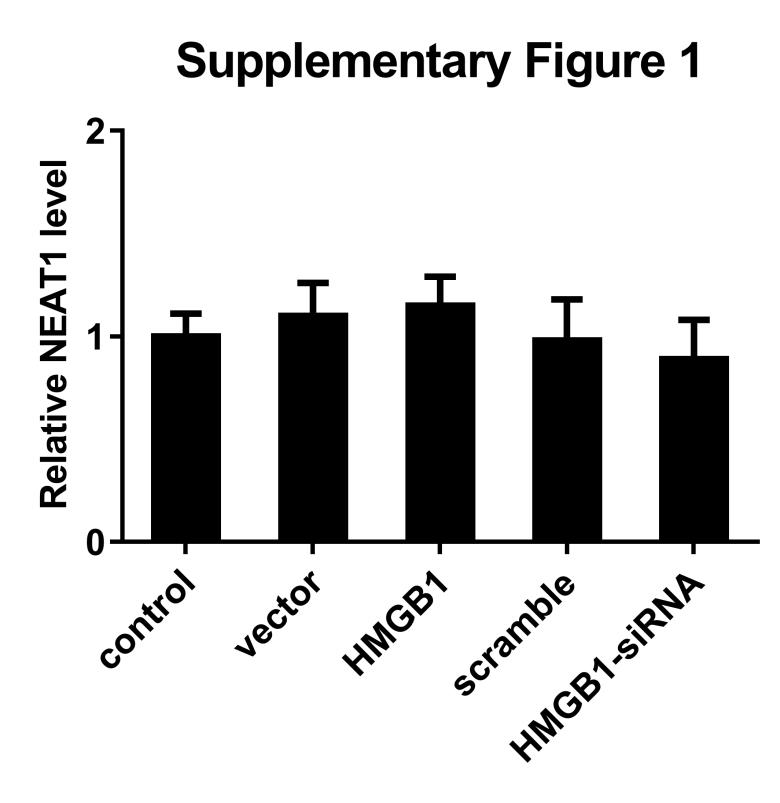

Supplement: Supplementary Materials — Supplementary Figure 1: NEAT1 levels were detected using RT-PCR. Cells were transfected with HMGB1 overexpression vector or HMGB1-siRNA. ∗P < 0.05 vs. the control group. [file 8019467.f1.jpg]
